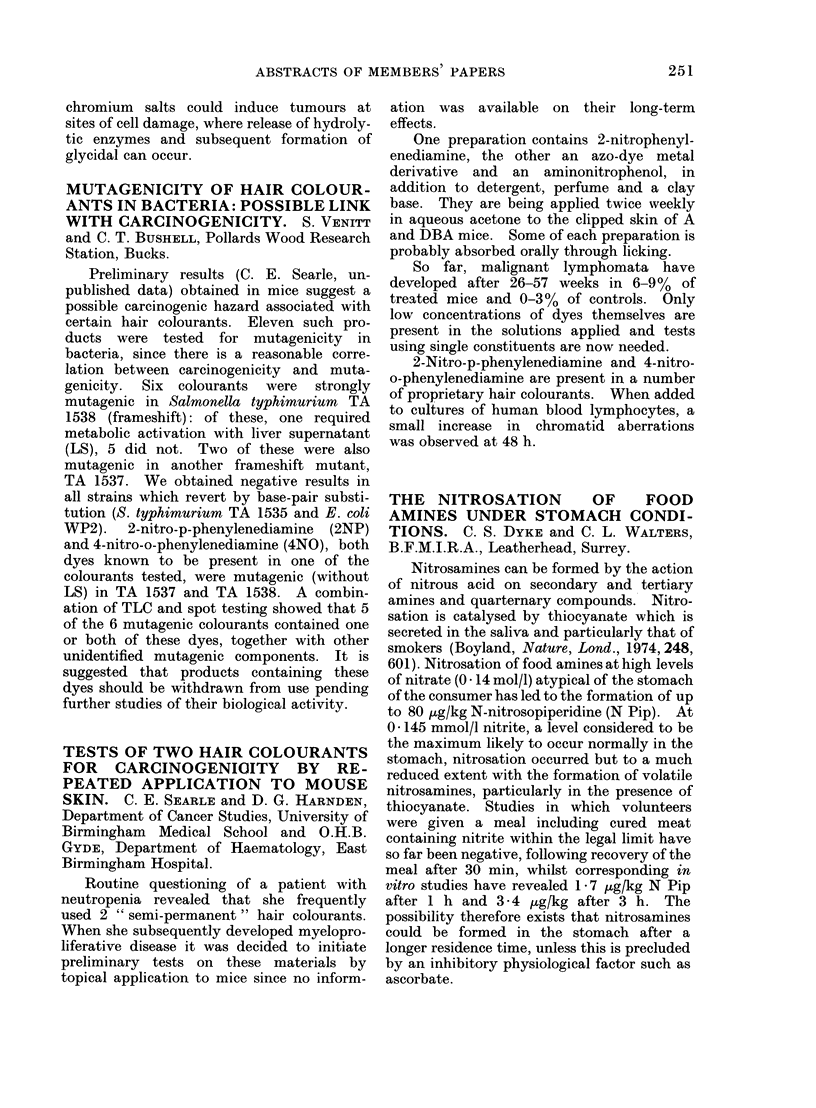# Proceedings: Mutagenicity of hair colourants in bacteria: possible link with carcinogenicity.

**DOI:** 10.1038/bjc.1975.191

**Published:** 1975-08

**Authors:** S. Venitt, C. T. Bushell


### MUTAGENICITY OF HAIR COLOURANTS IN BACTERIA: POSSIBLE LINK WITH CARCINOGENICITY.

S. VENITT and C. T. BUSHELL, Pollards Wood Research Station, Bucks.

Preliminary results (C. E. Searle, unpublished data) obtained in mice suggest a possible carcinogenic hazard associated with certain hair colourants. Eleven such products were tested for mutagenicity in bacteria, since there is a reasonable correlation between carcinogenicity and mutagenicity. Six colourants were strongly mutagenic in *Salmonella typhimurium* TA 1538 (frameshift): of these, one required metabolic activation with liver supernatant (LS), 5 did not. Two of these were also mutagenic in another frameshift mutant, TA 1537. We obtained negative results in all strains which revert by base-pair substitution (*S. typhimurium* TA 1535 and *E. coli* WP2). 2-nitro-p-phenylenediamine (2NP) and 4-nitro-o-phenylenediamine (4NO), both dyes known to be present in one of the colourants tested, were mutagenic (without LS) in TA 1537 and TA 1538. A combination of TLC and spot testing showed that 5 of the 6 mutagenic colourants contained one or both of these dyes, together with other unidentified mutagenic components. It is suggested that products containing these dyes should be withdrawn from use pending further studies of their biological activity.